# The relationship between body mass index and postoperative delirium

**DOI:** 10.1002/brb3.2534

**Published:** 2022-03-15

**Authors:** Xiyuan Deng, Peijuan Qin, Yanan Lin, He Tao, Fanghao Liu, Xu Lin, Bin Wang, Yanlin Bi

**Affiliations:** ^1^ Department of Anesthesiology Qingdao Municipal Hospital Affiliated to Qingdao University Qingdao China; ^2^ Department of Anesthesiology Weifang Medical University Weifang China; ^3^ Department of Anesthesiology Dalian Medical University Dalian China

**Keywords:** body mass index, cerebrospinal fluid, mediation analysis, postoperative delirium, tau proteins

## Abstract

**Purpose:**

We aimed to investigate the relevance of body mass index (BMI) to postoperative delirium (POD), and to test whether the influences of BMI on POD were mediated by cerebrospinal fluid (CSF) biomarkers.

**Patients and methods:**

Our study recruited 682 and 761 cognitively intact individuals from the perioperative neurocognitive disorder risk factor and prognosis (PNDRFAP) study and the perioperative neurocognitive disorder and biomarker lifestyle (PNDABLE) study, respectively. The incidence of POD was evaluated by using Confusion Assessment Method (CAM), and POD severity was measured by using the Memorial Delirium Assessment Scale (MDAS). Logistic regression was used to analyze the relationship between BMI and POD. The levels of Aβ40, Aβ42, T‐tau, and P‐tau in preoperative CSF were measured by enzyme‐linked immune‐sorbent assay (ELISA) in the PNDABLE study. Mediation analysis with 5000 bootstrapped iterations was used to explore the mediation effects.

**Results:**

In the PNDRFAP study, the incidence of POD was 16.3%, with logistic regression analysis showing that BMI (odds ratio [OR] = 0.900, 95% confidence interval [CI] 0.823–0.985, *p* = .022) is a protective factor of POD. In the PNDABLE study, the incidence of POD was 18.7%, and regression analysis confirmed that BMI (OR = 0.832, 95% CI 0.761–0.910, *p* < .001) is a protective factor of POD, while T‐tau (OR = 1.005, 95% CI 1.003–1.006, *p* < .001) and P‐tau (OR = 1.037, 95% CI 1.024–1.050, *p* < .001) were risk factors of POD. Mediation analyses revealed that the association between BMI and POD was partially mediated by T‐tau (proportion: 36%) and P‐tau (proportion: 24%).

**Conclusion:**

Higher BMI mediated protective effects on POD through CSF biomarkers (T‐tau and P‐tau).

## Graphical abstract

We investigate the relevance of BMI to POD and preoperative cerebrospinal fluid biomarkers were used for mediated analysis.

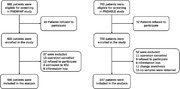

## 1 INTRODUCTION

Postoperative delirium (POD) is a common complication with cognitive impairment in patients after surgery. The overall incidence rates range between 10% and 60% (Dezube et al., 2020). It belongs to neurocognitive impairment that may occur after any type of surgical procedure. Hence, it has a significant impact on the prognosis of patients, especially elderly patients. Some studies speculated that POD may cause cognitive dysfunction through a mechanism similar to Alzheimer's disease (AD) (Fong et al., 2021; Racine et al., 2017), and some even believed that POD represents the early lesions of AD and accelerates the progression of AD (Fong et al., 2019), which poses a great threat to the quality of elderly life. Therefore, it is of great importance to identify the risk factors and elucidate pathophysiological mechanisms, and encourage patients to adjust their lifestyle and habits for the prevention of POD and AD.

Body mass index (BMI) is an internationally used index to evaluate obesity and health. The accumulation of fat in human body becomes more pronounced with BMI rises. A high BMI is closely associated with adverse events such as hypertension, hyperlipidemia, and insulin resistance (Haslam & James, 2005), which are thought to lead to cognitive dysfunction in a variety of ways. However, this view is also challenged by many other studies that show the opposite. Recently, a longitudinal study pointed out that a higher BMI in late‐life decreased the risk of AD, and the process may be driven by cerebrospinal fluid‐related biomarkers (Aβ, Tau) (Sun et al., 2020). Interestingly, several studies have found that delirium pathophysiology is similar to AD (Fong et al., 2021; Racine et al., 2017), as Aβ1‐40 and Aβ1‐42 levels were closely associated with POD (Ji et al., 2013; Rolandi et al., 2018). Indeed, the relevance of abnormal phosphorylation of tau protein to the occurrence and development of POD is widely acknowledged (Ji et al., 2018). However, there have been no reports in previous literature on whether BMI plays a similar role in the pathogenesis of POD. Given the great regulate of BMI, even a weak association with neurocognitive disorders such as AD or POD may lead to a high attributional risk, which has significant implications for public health.

Thus, we aimed to investigate the relationship between BMI and POD, to test whether the influences of BMI on delirium were mediated by POD core pathology. All the analyses were conducted based on the perioperative neurocognitive disorder risk factor and prognosis (PNDRFAP) study and perioperative neurocognitive disorder and biomarker lifestyle (PNDABLE) study.

## 2 PATIENTS AND METHODS

### 2.1 Participants

A total of 682 Han Chinese patients who were planned to undergo laparoscopic colorectal cancer resection under general anesthesia between February 2019 and May 2020 were selected from the PNDRFAP study, and a total of 761 Han Chinese patients who were planned to undergo knee or hip arthroplasty under combined spinal‐epidural anesthesia between February 2020 and August 2021 were selected from the PNDABLE study. PNDRFAP is a large cohort study conducted in 2019 to analyze the risk factors of perioperative neurocognitive impairment in the Han population in northern China for the early diagnosis and prevention of the disease. PNDABLE is also a large cohort study conducted in 2018 to analyze the risk factors and biomarkers of perioperative neurocognitive impairment in the Han population in northern China. The PNDRFAP study only included patients undergoing general anesthesia, while the PNDABLE study included only patients undergoing combined spinal and epidural anesthesia and collected preoperative cerebrospinal fluid for analysis of biomarkers. There was no patient overlap between the two studies. These two trials were carried out at Qingdao Municipal Hospital in Shandong Province, China. They were registered in the Chinese Clinical Research Registry (Clinical Registration Number of PNDRFAP: ChiCTR2000033639, PNDABLE: ChiCTR2000033439) and approved by the Ethics Committee of Qingdao Municipal Hospital, and informed consent was obtained from the patients.

In the PNDRAFP study, we included the following subjects: (1) the patients aged 40–90 years old, (2) American Society of Anesthesiologists physical status (ASA) Ⅰ–II, (3) the patients having intact preoperative cognitive function without communication disorders, and (4) the patients having sufficient education to complete the preoperative neuropsychological tests. Exclusion criteria were as follows: (1) Mini‐Mental State Examination (MMSE) scores of 23 or less, (2) ASA III or higher level, and (3) serious psychological disorders or deafness.

In the PNDABLE study, we included the following subjects: (1) the patients aged 40–90 years old, (2) ASA physical status Ⅰ–II, (3) the patients having intact preoperative cognitive function without communication disorders, and (4) the patients having sufficient education to complete the preoperative neuropsychological tests. Exclusion criteria were as follows: (1) MMSE scores of 23 or less, (2) ASA III or higher level, (3) serious psychological disorders, (4) severe systemic diseases that may affect related biomarkers in cerebrospinal fluid or blood, including but not limited to malignant tumors, (5) familial genetic diseases, and (6) coagulation dysfunction (possibly due to the long‐term use of anticoagulants).

### 2.2 Cognitive measurements

The same cognitive measurements were utilized in the two studies. Specifically, we used the MMSE to evaluate the basic cognitive level of the patients 1 day before surgery, and the Confusion Assessment Method (CAM) to evaluate the postoperative cognitive level at 9:00—10:00 am and at 2:00—3:00 pm twice a day on days 1—7 (or before discharge) by an anesthesiologist postoperatively. The diagnostic criteria for POD were as follows: (1) acute changes and repeated fluctuations in the state of consciousness, (2) lack of attention, (3) disorganized thinking, and (4) alterations in the level of consciousness. CAM was determined to be positive if both (1) and (2) were present on any day, and at the same time, either (3) or (4) was met. Based on the assessment results, the patients were divided into POD group and non‐POD group. The POD severity was assessed using the Memorial Delirium Assessment Scale (MDAS).

### 2.3 Anesthesia and surgery

All the patients in the two trials did not take any medication preoperatively, and surgery was performed by the same team of surgeons. After the patients entered the operating room, peripheral veins were opened, and electrocardiography (ECG), pulse blood oxygen saturation monitoring, and noninvasive arterial pressure measurements were routinely conducted.

Patients in the PNDRFAP database received general anesthesia as follows: 0.2–0.5 μg/kg sufentanil, 0.15–0.2 mg/kg cisatracurium, and 0.15–0.3 mg/kg etomidate were used for induction, 0.2–0.5 μg/kg/h dexmedetomidine was continuously pumped intraoperatively and stopped 30 min before surgery ended. Continuous pumping of 0.25–2 μg/kg/min remifentanil maintained analgesia, and cis‐atracurium was added intermittently every 40 min after induction and stopped 1 h before surgery ended. Sevoflurane supplementation was inhaled 0.5%–3% depending on the depth of anesthesia.

Patients in the PNDABLE database received spinal‐epidural anesthesia which was performed in the lateral decubitus under L3‐4 space. After successful puncture, 2.0–2.5 ml 0.67% ropivacaine was injected into the subarachnoid space, and then 3‐5 ml 0.375% ropivacaine was added into the epidural catheter according to actual needs to maintain the level of anesthesia at T8–S5. During the operation, vasoactive drugs were used moderately to maintain the vital signs of the patients at a stable level. Every patient was treated with a patient‐controlled intravenous analgesia pump (tropisetron 5 mg+ butorphanol tartrate injection 10 mg, diluted to 100 ml with normal saline at a rate of 2 ml/h) for 48 h postoperatively. After the operation, the patient was sent to the PACU (Postanesthesia Care Unit), observed for 30 min, and sent back to ward if there was no abnormality. The duration of surgery, duration of anesthesia, intraoperative blood loss, and fluid input were recorded.

### 2.4 Measurements of cerebrospinal fluid sampling

Cerebrospinal fluid samples were taken from patients in the PNDABLE database. After successful spinal‐epidural anesthesia puncture, 2 ml of cerebrospinal fluid was collected in 10 ml polypropylene tubes and sent to the laboratory within 2 h. The cerebrospinal fluid (CSF) samples were immediately centrifuged at 2000 *g* at room temperature for 10 min and then stored at −80°C for further analysis. The levels of Aβ40, Aβ42, total Tau (t‐Tau), and phosphorylated Tau (p‐Tau) in CSF were determined by enzyme‐linked immunosorbent assays (ELISAs) using INNOTEST (Fujirebio Europe N.V.) on the microplate reader (Thermo Scientific MultiskanMK3). All CSF samples were randomly distributed on the same batch of plates. All experimental procedures were performed by researchers who were blinded to patient information.

### 2.5 Sample size estimation

The preliminary test in the PNDRFAP study found that eight covariates (age, education, BMI, MMSE, albumin, duration of surgery, duration of anesthesia, and estimated blood loss) were expected to enter the logistic regression. The POD incidence was 14.7%, and the loss of follow‐up rate was assumed to be 20%, so the required sample size was calculated to be 680 cases (8 × 10 ÷ 0.147 ÷ 0.8 = 680). The preliminary test in the PNDABLE study found that nine covariates (age, BMI, MMSE, Diabetes, CHD, Aβ40, Aβ42, T‐tau, P‐tau) were expected to enter the logistic regression. The POD incidence was 14.7%, and the loss of follow‐up rate was assumed to be 20%, so the required sample size was calculated to be 765 cases (9 × 10 ÷ 0.147 ÷ 0.8 = 765).

### 2.6 Statistical analysis

Characteristics of the participants were represented as the mean ± SD, the median and interquartile range (IQR, 25–75 percentiles), or a percentage (%). We used the Kolmogorov–Smirnov test to test the normality of all variables. For variables with normal distribution, independent sample *t*‐test was used to compare the difference between groups. When the continuous variables were non‐normally distributed, nonparametric methods were adopted. Mann–Whitney *U* test was used to compare the difference between groups, and *χ* ^2^ test to compare categorical variables and the incidence of POD was expressed as a percentage.

Significant variables were included in univariate regression analysis. Then, multivariable logistic regression analysis was performed after adjusted for age, sex, education, and MMSE score (in both PNDRFAP and PNDABLE databases). To explore whether the relationship between BMI and POD was mediated by POD pathology, the mediation analysis was fitted according to the method proposed by Baron and Kenny. The significance was determined by 5000 bootstrap iterations using the mediation effect. *p* < .05 was considered statistically significant.

In addition, a sensitivity analysis was performed to assess the results stability. It was carried out as follows. First, we analyzed whether the association would change if only individuals aged over 65 at the baseline were selected. Second, we added more covariates, such as hypertension, diabetes, coronary heart disease, and history of smoking and drinking.

The data were analyzed using SPSS version 23.0 (SPSS, Inc, Chicago, Illinois, USA), GraphPad Prism version 7.0 (GraphPad Software, Inc., LaJolla, CA, USA), and Stata MP16.0 (Solvusoft Corporation, Inc, Chicago, Illinois, USA).

## 3 RESULTS

### 3.1 Participant characteristics

**FIGURE 1:**
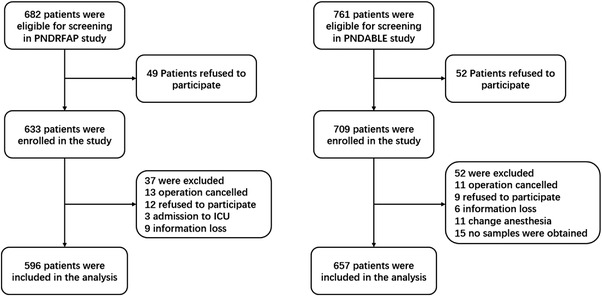
Flow diagram of the perioperative neurocognitive disorder risk factor and prognosis (PNDRFAP) study and the perioperative neurocognitive disorder and biomarker lifestyle (PNDABLE) study

As for PNDRFAP, we included 682 participants, of which 596 met the requirements of this study and 86 were excluded. The reasons for dropping out are shown in Figure 1. Of the enrolled patients, 97 subjects experienced POD within 7 days after operation or before discharge. The demographic and clinical data of the participants are summarized in Table 1. We found that the incidence of POD was 16.3%, and there was a statistically significant difference in BMI between the two groups (*p* < .05). After adjusted for age, sex, education, and MMSE score, the multivariable logistic regression analysis shows that BMI is a protective factor of POD (Table 2).

**TABLE 1:** Demographic and clinical characteristics of participants in the perioperative neurocognitive disorder risk factor and prognosis (PNDRFAP) study and the perioperative neurocognitive disorder and biomarker lifestyle (PNDABLE) study

	PNDRFAP			PNDABLE		
Participant features	POD (*n* = 97)	non‐POD (*n* = 499)	*p*‐Value*	POD (*n* = 123)	non‐POD (*n* = 534)	*p*‐Value*
Age, yr	76.3 ± 8.6	63.6 ± 10.4	.043*	74.3 ± 5.5	59.6 ± 8.1	<.001***
Female	46 (47.4)	214 (42.9)	.435	46 (30.1)	204 (35.6)	.918
Education, yr	5 (5, 9)	9 (8, 12)	<.001***	10 (7, 12)	10 (9, 12)	.141
BMI, kg/m^2^	23.9 ± 3.5	25.3 ± 3.6	.001**	24.0 ± 3.3	25.8 ± 3.7	<.001***
Coexisting diseases						
Hypertension	49 (50.1)	219 (43.9)	.265	49 (32.0)	174 (31.0)	.139
Diabetes	26 (26.8)	112 (22.4)	.359	34 (27.6)	74(13.9)	<.001***
CHD	22 (22.7)	102 (20.4)	.682	28 (22.8)	59 (11.0)	.001**
Stroke	—	—	—	11 (9.0)	20 (3.7)	.030*
MMSE	24.6 ± 1.0	26.9 ± 1.3	<.001***	27.8 ± 1.4	28.3 ± 1.7	.001**
Smoking history	36 (37.1)	176 (35.3)	.729	36 (29.3)	159 (29.8)	1.000
Drinking history	37 (38.1)	150 (30.0)	.121	38 (30.9)	191 (35.8)	.345
Preoperative laboratory tests						
Albumin, g/L	36.4 ± 3.8	38.7 ± 3.4	.036**	—	—	—
Glucose, mmol/L	6.0 ± 2.0	5.8 ± 2.0	.071	5.7 ± 1.5	5.5 ± 1.4	.308
Potassium, mmol/L	4.0 ± 0.4	3.9 ± 0.4	.899	—	—	—
Duration of surgery, h	120 (85, 205)	110 (65, 165)	.013*	120 (110, 130)	120 (110, 130)	.344
Duration of anesthesia, h	180 (135, 267)	155 (105, 220)	.002**	140 (130, 160)	140 (130, 160)	.679
Intraoperative fluid, ml	1100 (1000, 1850)	1100 (1000, 16000)	.038*	800 (800, 900)	800 (800, 900)	.440
Estimated blood loss, ml	50 (20, 200)	20 (10, 100)	<.001***	120 (110, 130)	120 (110, 130)	.808

Abbreviations: BMI, body mass index; CHD, coronary heart disease; MMSE, Mini‐Mental State Examination; POD, postoperative delirium.

* *p*‐Value < .05.

** *p*‐Value < .01.

*** *p*‐Value < .001.

**TABLE 2:** Logistic regression analysis and sensitivity analysis in the perioperative neurocognitive disorder risk factor and prognosis (PNDRFAP) study

	Model 1		Model 2		Model 3		Model 4	
	OR (95% CI)	*p*‐Value	OR (95% CI)	*p*‐Value	OR (95% CI)	*p*‐Value	OR (95% CI)	*p*‐Value
BMI, kg/m^2^	0.900 (0.845–0.958)	.001	0.900 (0.823–0.985)	.022	0.890 (0.802–0.987)	.028	0.888 (0.797–0.989)	.031
Albumin, g/L	0.834 (0.782–0.890)	<.001	0.937 (0.853–1.029)	.175	1.012 (0.906–1.130)	.839	0.994 (0.889–1.110)	.909
Duration of surgery, h	1.001 (0.999–1.002)	.240	‐ ‐ ‐ ‐	–	‐ ‐ ‐ ‐	–	‐ ‐ ‐ ‐	–
Duration of anesthesia, h	1.002 (1.000–1.004)	.017	1.002 (0.999–1.004)	.192	1.002 (0.999–1.005)	.132	1.002 (0.999–1.005)	.104
Intraoperative fluid, ml	1.000 (1.000–1.000)	.193	‐ ‐ ‐ ‐	–	‐ ‐ ‐ ‐	–	‐ ‐ ‐ ‐	–
Estimated blood loss, ml	1.003 (1.001–1.004)	.002	1.001 (0.998–1.003)	.643	1.000 (1.997–1.003)	.931	1.001 (0.997–1.003)	.943

*Note*: Model 1: the unadjusted logistic regression; Model 2: adjusted logistic regression, the adjustment factors include age, sex, education, and MMSE score; Model 3: first sensitivity analysis was based on selecting only individuals older than 65 years; Model 4: second sensitivity analysis was based on more covariables including hypertension, diabetes, coronary heart disease, and history of smoking and drinking.

Abbreviations: BMI, body mass index; CI, confidence interval; OR, odds ratio.

As for PNDABLE, we included 761 participants, of which 657 met the requirements of this study and 104 were excluded. The reasons for dropping out are shown in Figure 1. Of the enrolled patients, 123 subjects experienced POD within 7 days after operation or before discharge. The demographic and clinical data of the participants are summarized in Table 1. The incidence of POD was 18.7%. Adjusted multivariate regression shows that BMI is still a protective factor for POD (Table 2).

### 3.2 The relationship between CSF biomarkers and POD

**FIGURE 2:**
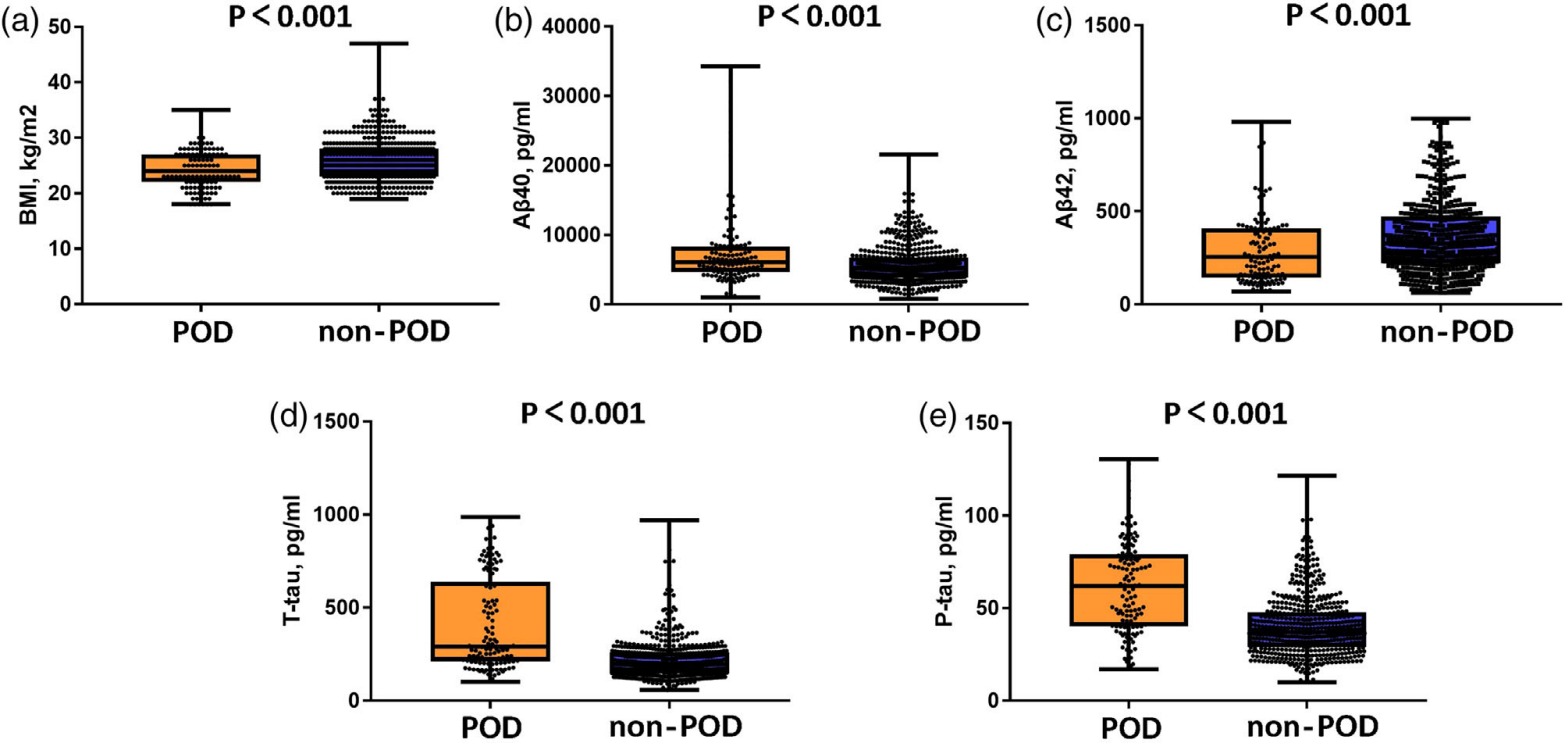
Expression of biomarkers in cerebrospinal fluid (CSF) of postoperative delirium (POD) patients and non‐POD controls

In addition to BMI, the concentrations of CSF biomarkers (Aβ40, Aβ42, T‐tau, and P‐tau) were compared between POD patients and non‐POD patients before operation. Mann–Whitney test showed that the CSF levels of Aβ40, P‐tau, and T‐tau in patients with delirium were significantly higher than those in patients without delirium. However, the CSF levels of Aβ42 in POD patients were significantly lower than those in non‐POD patients (Figure 2). Unadjusted regression shows that Aβ40 (odds ratio [OR] = 1.000 95% confidence interval [CI] = 1.000–1.000) has no significance, and adjusted multivariate regression shows that Aβ42 (OR = 1.000 95% CI = 0.998–1.001) has no significance. T‐tau (OR = 1.005 95% CI = 1.003–1.006) and P‐tau (OR = 1.037 95% CI = 1.024–1.050) maintained great significance and were risk factors for POD (Table 2).

### 3.3 Causal mediation analyses

**FIGURE 3:**
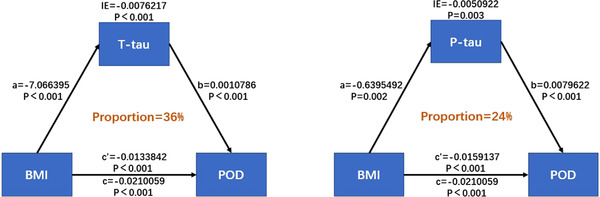
Mediation analyses

The multivariate regression in the PNDABLE study shows that BMI, T‐tau, and P‐tau were positively correlated with POD; therefore, we speculate that BMI is not only a protective factor of POD but may also regulate the occurrence of POD through Tau pathology. We further explored whether T‐tau and P‐tau could mediate the effect of BMI on POD. The mediation analysis showed that the relationship between BMI and POD was mediated by T‐tau (the proportion of intermediaries is about 36%–37%) and P‐tau (the proportion of intermediaries is about 23%–24%) (Figure 3). The effect was considered partial mediation.

### 3.4 Sensitivity analysis

To verify the stability of the results, we performed sensitivity analyses on both PNDRFAP and PNDABLE studies, using two models which were based on higher ages and more covariables, respectively. BMI remained stable across four sensitivity analyses in the two studies, and T‐tau and P‐tau in the PNDABLE study also remained stable (Table 3). To sum up, the sensitivity analysis has showed that the results were stable.

**TABLE 3:** Logistic regression analysis and sensitivity analysis in the perioperative neurocognitive disorder and biomarker lifestyle (PNDABLE) study

	Model 1		Model 2		Model 3		Model 4	
	OR (95% CI)	*p*‐Value	OR (95% CI)	*p*‐Value	OR (95% CI)	*p*‐Value	OR (95% CI)	*p*‐Value
BMI, kg/m^2^	0.845 (0.801–0.910)	<.001	0.832 (0.761–0.910)	<.001	0.827 (0.755–0.906)	<.001	0.831 (0.758–0.911)	<.001
Diabetes	2.375 (1.491–3.781)	<.001	1.138 (0.622–2.083)	.674	‐ ‐ ‐ ‐	–	‐ ‐ ‐ ‐	–
CHD	2.373 (1.438–3.915)	.001	0.764 (0.383–1.523)	.445	‐ ‐ ‐ ‐	–	‐ ‐ ‐ ‐	–
Stroke	2.524 (1.176–5.417)	.017	0.861 (0.305–2.431)	.778	‐ ‐ ‐ ‐	–	‐ ‐ ‐ ‐	–
Aβ40, pg/ml	1.000 (1.000–1.000)	<.001	‐ ‐ ‐ ‐	–	‐ ‐ ‐ ‐	–	‐ ‐ ‐ ‐	–
Aβ42, pg/ml	0.998 (0.997–0.999)	.003	1.000 (0.998–1.001)	.645	‐ ‐ ‐ ‐	–	‐ ‐ ‐ ‐	–
T‐tau, pg/ml	1.006 (1.005–1.008)	<.001	1.005 (1.003–1.006)	<.001	1.004 (1.002–1.006)	<.001	1.005 (1.003–1.006)	<.001
P‐tau, pg/ml	1.053 (1.041–1.064)	<.001	1.037 (1.024–1.050)	<.001	1.036 (1.023–1.050)	<.001	1.038 (1.025–1.052)	<.001

*Note*: Model 1: the unadjusted logistic regression; Model 2: adjusted logistic regression, the adjustment factors include age, sex, education, and MMSE score; Model 3: first sensitivity analysis was based on selecting only individuals older than 65 years; Model 4: second sensitivity analysis was based on more covariables including hypertension, diabetes, coronary heart disease, and history of smoking and drinking.

Abbreviations: BMI, body mass index; CI, confidence interval; CHD, coronary heart disease; OR, odds ratio.

## 4 DISCUSSION

In this study, we used two databases to evaluate the association between BMI and POD. The results show that BMI is a protective factor of POD, and two CSF biomarkers were selected as mediators. This finding supports the “obesity paradox” which means that higher BMI can reduce the risk of POD.

BMI is a widely recognized measure of body weight and health that is defined as weight in kilograms divided by the square of the height in meters. High BMI is closely associated with adverse events including cognitive dysfunction (Anstey et al., 2010; Wu et al., 2011), but the correlation between BMI and POD has not been fully characterized. Given the adjustability of BMI and the high incidence of POD, even a weak correlation between the two could have a significant impact on the prevention of POD. In our analysis of the PNDRFAP database, we found that higher BMI was a protective factor for POD and exhibited great stability. To verify this result, we analyzed the PNDABLE database and reached the same conclusion, which is consistent with recent research on BMI and AD (Sun et al., 2020). POD and AD are closely related to pathogenesis (Fong et al., 2019); therefore, we speculated that BMI might affect POD through a pathophysiological mechanism similar to AD. BMI may exert both positive and negative effects on POD, AD, and other neurocognitive disorders in a context‐dependent manner, which could explain the inconclusive correlation between BMI and POD observed so far. Our study supports the obesity paradox (Frisardi et al., 2020), but this theory has been highly controversial as the negative effects of obesity are obvious, for example, insulin resistance (Kleinridders et al., 2017; Waki et al., 2014), oxidative stress (Rodriguez‐Casado et al., 2013), lepin (Burguera et al., 2009; Gorrini et al., 2000), and adiponectin (Lieb, 2016). Hence, it remains to be determined whether the overall impact will be more beneficial or harmful. Research on the obesity paradox has focused on cardiovascular disease, and research in the cognition field is sparse but equally controversial (Wang & Scherer, 2017). In an 8‐year prospective investigation of 1351 subjects, higher BMI was not associated with increased dementia risk (Monda et al., 2007). Another study suggests that weight gain is unrelated to cognitive performance (Buchman et al., 2021). Instead, a gain in bodyweight is disadvantageous when signaling illness or reduced physical activity, while it is beneficial when pointing to health recovery. Indeed, only respondents with preceding weight loss profited from small increases in BMI. In our study, although most of the patients included were overweight, only a few were obese. In addition, our study features a large age range that includes a cohort of middle‐aged subjects, which could offset the negative effects of obesity on the elderly to some extent and magnify the cognitive benefits of being overweight. This could explain why our results support the obesity paradox.

Preoperative CSF was collected in the PNDABLE study. CSF is thought to accurately reflect changes in the brain; hence biomarkers in CSF can be used to assess neuropathologies in living individuals (Kronschnabl et al., 2020). In particular, Tau is a microtubule‐associated protein present in the axon of neurons which is essential for microtubule stabilization and axonal transport. Phosphorylation of tau is believed to causes it to self‐aggregate, and neurofibrillary tangles (NFTs) are intraneuronal aggregates that are mainly composed of helical filaments of hyperphosphorylated tau (Guo et al., 1986; Han et al., 1975; Weingarten et al., 2017). CSF T‐tau is suggested to reflect the severity of axonal degeneration (Blennow & Hampel, 2019; Bos et al., 2016; Grundke‐Iqbal et al., 2003) and P‐tau the tangle pathology (Blennow & Zetterberg, 2018; Mattsson et al., 2016; Olsson et al., 2018). Moreover, accumulation of extracellular deposits of abnormally folded amyloid‐β (Aβ) peptides (amyloid plaques) and intraneuronal inclusions of NFTs are considered characteristics of AD. Over the last 2 decades, research criteria for AD have integrated biomarkers of amyloid plaques and NFTs (Aβ, T‐Tau, and p‐Tau) in the diagnostic process (Henriques et al., 2019). In our study, there were significant differences in Aβ40, Aβ42, T‐tau, and P‐tau between the POD and non‐POD groups. This is consistent with recent studies on POD, demonstrating that CSF biomarkers of AD can play a similar role in POD. Surprisingly, the adjusted regression analysis showed that Aβ40 and Aβ42 were not significantly correlated with POD, while T‐tau and P‐tau were still positively correlated with POD. Hence, they can mediate the effect of BMI on POD. On the other hand, our findings support the notion that Tau pathology and amyloid deposition may be independent of each other, which is consistent with previous studies (Cunningham et al., 2014). In fact, increasing evidence has shown that Tau pathology may play a more significant role than Aβ protein deposition, as some studies found only minor effects caused by Aβ on cognition in normal older adults (Baker et al., 2017; Bloom, 2017; Duke Han et al., 2018), and that Aβ deposition has to be accompanied with tauopathy in order to have considerable impact on cognition (Desikan et al., 2016; Jansen et al., 2019; Soldan et al., 2012; Soldan et al., 2018). In addition, previous studies have found that some novel biomarkers, such as NFL, YKL‐40, and FABP3, were specifically correlated with and clustered with T‐tau and P‐tau, but not with Aβ42 (Vos & Visser, 2020). As Tau pathology is the result of long‐term progression, while BMI is a stable long‐term regulatory factor, we speculate that BMI can delay the progression of Tau protein pathology through a variety of mechanisms, thus reducing the risk of POD.

Our study has some limitations. First, there are few reports on POD and BMI in the past, so the results of our research need to be confirmed by multicenter large sample studies. Second, BMI has some limitations as a standard for classification of obesity, and we suggest that abdominal circumference and body fat rate should be included as the new evaluation criteria. Finally, due to the limited sample size, only a few obese patients were included in our research. In fact, BMI was analyzed as a noncategorical variable, and low‐weight patients were excluded, which could skew the results. Hence, future studies with a larger sample are desired to analyze categorical variables.

## 5 CONCLUSION

Our research reveals the effect of BMI on POD, supports the obesity paradox, and has, for the first time, used mediation analysis to explain the relationship between BMI and CSF biomarkers, laying the foundation for follow‐up research. We have thus provided a new insight into the prevention of POD, which may change the concept of lifestyle and weight control.

## CONFLICT OF INTEREST

The authors declare no conflict of interest.

### PEER REVIEW

The peer review history for this article is available at https://publons.com/publon/10.1002/brb3.2534.

## Data Availability

The datasets used and/or analyzed during the current study are available from the corresponding author on reasonable request.
